# Current Understanding of the Role of T Cells in Chikungunya, Dengue and Zika Infections

**DOI:** 10.3390/v14020242

**Published:** 2022-01-25

**Authors:** Maheshi Mapalagamage, Daniela Weiskopf, Alessandro Sette, Aruna Dharshan De Silva

**Affiliations:** 1 Department of Zoology and Environment Sciences, Faculty of Science, University of Colombo, Colombo 00700, Sri Lanka; maheshi@zoology.cmb.ac.lk; 2 Center for Infectious Disease and Vaccine Research, La Jolla Institute for Immunology (LJI), La Jolla, CA 92037, USA; dweiskopf@lji.org (D.W.); alex@lji.org (A.S.); 3 Department of Medicine, Division of Infectious Diseases and Global Public Health, University of California San Diego (UCSD), La Jolla, CA 92037, USA; 4 Department of Paraclinical Sciences, Faculty of Medicine, General Sir John Kotelawala Defence University, Colombo 10390, Sri Lanka

**Keywords:** Chikungunya, Dengue, Zika, cross-reactivity, CD4 T cells, CD8 T cells, vaccines, T cell epitopes

## Abstract

Arboviral infections such as Chikungunya (CHIKV), Dengue (DENV) and Zika (ZIKV) are a major disease burden in tropical and sub-tropical countries, and there are no effective vaccinations or therapeutic drugs available at this time. Understanding the role of the T cell response is very important when designing effective vaccines. Currently, comprehensive identification of T cell epitopes during a DENV infection shows that CD8 and CD4 T cells and their specific phenotypes play protective and pathogenic roles. The protective role of CD8 T cells in DENV is carried out through the killing of infected cells and the production of proinflammatory cytokines, as CD4 T cells enhance B cell and CD8 T cell activities. A limited number of studies attempted to identify the involvement of T cells in CHIKV and ZIKV infection. The identification of human immunodominant ZIKV viral epitopes responsive to specific T cells is scarce, and none have been identified for CHIKV. In CHIKV infection, CD8 T cells are activated during the acute phase in the lymph nodes/blood, and CD4 T cells are activated during the chronic phase in the joints/muscles. Studies on the role of T cells in ZIKV-neuropathogenesis are limited and need to be explored. Many studies have shown the modulating actions of T cells due to cross-reactivity between DENV-ZIKV co-infections and have repeated heterologous/homologous DENV infection, which is an important factor to consider when developing an effective vaccine.

## 1. Introduction

**Figure 1:**
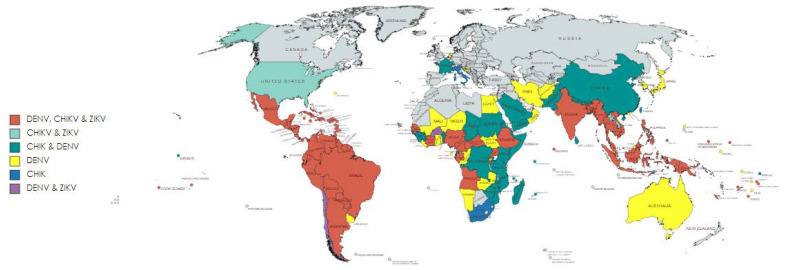
Global distribution of CHIKV, DENV and ZIKV infections. This map shows the global transmission of CHIKV, DENV and ZIKV. The map was created using the free online tool (https://mapchart.net/detworld.html; accessed on 17 December 2021) and inspired by [2]. The figure was created based on the data provide be following websites (accessed on 17 December 2021): https://www.cdc.gov/dengue/areaswithrisk/around-the-world.html; https://www.gov.uk/guidance/zika-virus-country-specific-risk#atoz; https://www.cdc.gov/chikungunya/geo/index.html.

Chikungunya virus (CHIKV), Dengue virus (DENV) and Zika virus (ZIKV) are widely distributed arboviruses transmitted through *Aedes* mosquitos, causing a massive disease burden in tropical and subtropical areas of the world. CHIKV is an alphavirus (Togaviridae family), and DENV and ZIKV are flaviviruses (Flaviviridae family) [1]. These virus infections are endemic in largely overlapping geographical regions. They initially emerged in Africa, where CHIKV, DENV and ZIKV infections are common (Figure 1).

DENV and ZIKV are enveloped viruses with a single-stranded, 10.7 kb, positive-sense RNA genome, translated as a single polyprotein cleaved into three structural proteins (C, prM/M, E) and seven nonstructural proteins (NS1, NS2A, NS2B, NS3, NS4, NS5A and NS5B) by both viral and host proteases [3,4]. CHIKV encodes for three structural (C, E1, E2) and four nonstructural proteins (nsP1-4) [5]. The commonly observed signs and symptoms of CHIKV, DENV and ZIKV infections include fever, headache, rash, arthralgia and myalgia [6].

Despite a wealth of research conducted globally across the past few decades, there are limited commercially available antiviral drugs or vaccines for these infections. The treatments usually involve analgesics, anti-inflammatory drugs and supportive care. Dengvaxia^®^ by Sanofi Pasteur is considered the first licensed DENV vaccine. However, recent studies have questioned the efficacy and raised safety concerns of Dengvaxia^®^ because vaccine recipients who were not exposed to previous dengue had a higher risk of hospitalization due to severe dengue infection [7,8,9]. Apart from Dengvaxia, there are 16 dengue vaccine candidates in clinical and pre-clinical trials [10]. Nearly 20 ZIKV vaccine candidates are being tested with different approaches, and three different vaccines are undergoing clinical trials: 1. Inovio (GLS5700), targeting prME of ZIKV [11]; 2. NIAID: VRC-ZKADNA085-00-VP (VRC 5288); and 3. VRC-ZKADNA090-00-VP (VRC 5283) [12]. More than 18 CHIKV vaccine candidates are being studied globally, including advance formulations such as TSI-GSD-218, which completed the phase II trial, and the VRCCHKVLP059-00-VP, which is at phase II [13,14].

The issues of potential DENV and ZIKV cross-reactivity, as well as multiple heterologous and homologous DENV infections skewing T-cell responses are of relevance for understanding the patterns of natural immunity and the development of diagnostic tests and vaccines. Cross-reactivity at the serological and T cell levels has been reported in DENV, CHIKV and ZIKV infections. However, its impact on infection and disease has not been fully understood. Therefore, the role of T cells in CHIKV, DENV and ZIKV infection; the identification of specific viral epitopes by T cells; and the cross-reactivity between DENV and ZIKV and heterologous DENV infection are discussed below with the aim of understanding the importance of studying T cell immunity during vaccine design.

## 2. Epitope Identification of CHIKV, DENV and ZIKV; Progression and Knowledge Gap

As T cells play a pivotal role in immunity and pathogenesis, it is very important to identify the molecular epitopes recognized as a result of infection. In humans, the immunodominance of these epitopes can be characterized by their ability to bind HLA class I and II molecules, and the binding efficacy of these epitopes to T cell receptor (TCR). Not all peptides have the capacity to show high immunodominance. Therefore, it is imperative to study the most immunodominant peptides in the virus which could be considered as potential targets for epitope-based vaccination. In turn, this will allow for the definition of the phenotypes of T cells associated with natural immunity and vaccination, which can help to address how the nature of the epitopes and/or the nature of the responding T cells are influenced by multiple infections. Therefore, this section analyzes published data on the CHIKV, DENV and ZIKV epitopes identified by our team and others using the data available in the Immune Epitope Database (IEDB: www.IEDB.org, (accessed on 30th November 2021)). A previous review published by our team presented a descriptive analysis of human T cell response to DENV infection based on data specifically for DENV available in IEDB up to July 2019 [15].

Immunodominant epitopes for CHIKV, DENV and ZIKV were retrieved from the IEDB database on 30 November 2021 using the following search parameters; any epitope, positive assays only, organism: dengue virus (ID:12637)/Chikungunya virus (ID:37124)/Zika virus (ID:64320), no B cell assays, no MHC ligand assays, host: human (Homo Sapiens), disease: infectious.

Accordingly, for DENV, the IEDB listed 832 and 1364 DENV T cell epitopes identified in humans restricted by HLA class I and class II, respectively. A total 69 references were available in the IEDB database, of which 57 references were described in detail by Tian et al. (2018) [15]. An immunoinformatic study suggests the HTLWSNGVL and FTTNIWLKL epitope peptides in the NS1 protein sequence as potential agents for a multi-cytotoxic T lymphocyte (CTL) epitope vaccine [16].

**Figure 2:**
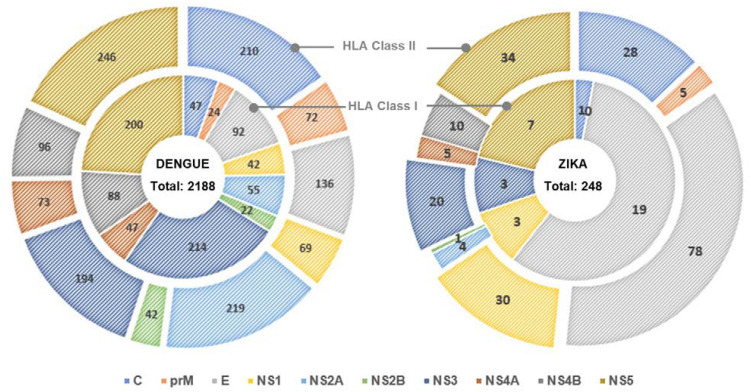
Number of immunodominant T cell epitopes against dengue (DENV) and Zika viruses (ZIKV). Data were retrieved from the IEDB (on 30 November 2021) corresponding to the HLA restriction (HLA class I: inner circle, HLA II: outer circle) and protein region.

Although a vast number of studies and reviews has been conducted for DENV, very limited references are available for epitope identification of ZIKV infection. As of November 2021, only 10 references were available in the IEDB human sample database. Seven studies focused on both CD4^+^ and CD8^+^ T cell responses [17,18,19,20,21,22,23], and three [24,25,26] studies focused on only CD4^+^ T cell responses [26]. In these studies, only 33 of HLA class I restricted and 215 of HLA class II restricted epitopes of ZIKV-T cell epitopes identified in humans were documented in the IEDB. Figure 2 provides a comparison of the number of epitopes recorded in IEDB with respect to HLA I and HLA II restriction in DENV and ZIKV corresponding to the protein region of the immunodominant epitope. The highest percentage of dominant epitopes of ZIKV responding to both HLA I (57.6%) and HLA II (36.3%) was present in the E protein region. In contrast, the highest percentage of dominant epitopes of DENV for HLA I and HLA II was present in the NS3 protein (25.7%) region and the NS5 protein region (18.1%), respectively. GLDFSDLYY-restricting HLA I was the only ZIKV epitope to appear independently in two references [17,22]. All other ZIKV epitopes were confirmed by only one study. The median number of assays performed for the immunodominant epitope identification of ZIKV for HLA I and HLA II was 5 and 10, respectively. However, only a median of one responding donor was present in the IEDB database for both HLA I and HLA II restriction.

The lack of identification of plausible immunodominant T cell epitopes against ZIKV shows the importance of conducting more experiments to study the immunodominance of ZIKV virus toward human T cell responses, which will be useful in future vaccine development.

In contrast to DENV and ZIKV, no human CHIKV T cell specific epitopes have been defined and published to date using in vitro or ex vivo models. However, bioinformatic and sub-genomic analysis were conducted using the E2 protein of CHIKV genome in Pakistani isolates. Epitope prediction for this study was performed using CTLPred and nHLAPred tools, and two highly immunogenic and conserved CHIKV epitopes restricted by HLA class I (STKDNFNVY and SRPQHGKEL) were found [27].

A comprehensive analysis of HLA class I and II restricted responses across all loci is needed for CHIKV and ZIKV. The analysis should address both the general population from endemic areas and patient populations associated with different severities of disease. Likewise, for CHIKV and ZIKV infections, the epitopes recognized after vaccination with experimental vaccines have not been systematically identified or validated. One recent study suggests an in-silico machine learning based approach for ZIKV T cell epitope prediction, which is useful for epitope-based peptide vaccination [28]. However, the lack of knowledge of CHIKV and ZIKV immunodominant epitopes hampers progress in the field, as the role of T cell responses in disease protection and immunopathology cannot be broadly evaluated, and the performance of different vaccines in terms of induction of immune responses in human remains undetermined.

## 3. Complex Role of T Cells in CHIKV, DENV and ZIKV

### 3.1. Understanding the Role of T Cells in CHIKV: Nescience and Opportunity for Research

**Figure 3:**
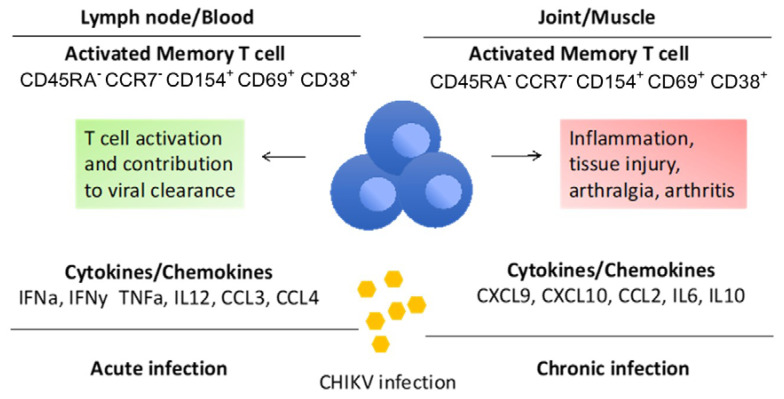
T cell response to CHIKV in acute and chronic infection.

Unlike other alphaviruses, CHIKV infection has higher clinical presentation, where 50–97% of infected patients can develop clinical symptoms. CHIKV infection is also associated with higher disease morbidity than mortality [5,29]. In addition, CHIKV has detrimental effects on tissues, partly mediated by an over-activated host immune system, with a robust immune response associated with type-I interferon (IFN) pathways. In the majority of patients, CHIKV replicates at very high levels in the blood (up to 10^10^ viruses/mL of blood) during the acute symptomatic phase. However, CHIKV replicates without any clinically apparent sequalae [30]. This is the acute recovery/resolving phase where the immune system plays an important role in viral clearance and T cells contribute to limit the spread of virus. In elderly patients where viremia is high and long-lasting, a chronic phase that can last from months to years is usually associated with marked inflammatory reaction, recurring arthralgia and arthritis [31]. Figure 3 represents how T cells respond during acute and chronic infection of CHIKV, releasing specific cytokines and chemokines into lymph nodes and tissues, respectively [5].

In human CHIKV infection, acute lymphopenia has been observed, and has been delineated as having <1000 lymphocytes/mL [30]. Studies have confirmed that CD8^+^ T cells predominate in the early stages of the disease, with CD4^+^ T cells mediating the adaptive response at later times of post infection [32,33]. During acute CHIKV infection, CD8^+^ T cells express CD69, CD107a, granzme B and perforin, which are responsible for killing the virus-infected cells by inducing cytolytic mechanisms [32,34,35,36]. However, studies have shown that having high antigen levels in the acute phase of infection could lead to the continuous presentation of CHIKV epitopes by antigen-presenting cells (APCs) to CD8^+^ T cells. This could exhaust these CD8^+^ T cells, resulting in low efficiency of eliminating the infected cells. In turn, this may lead to chronic CHIKV infection, with lower levels of functioning CD8^+^ T cells [35,37]. In contrast, studies have shown that CD4^+^ T cells activate during the chronic phase of CHIKV infection, inducing inflammation by producing proinflammatory cytokines. Excessive cellular infiltrates into the inflamed tissues, resulting in joint swelling [36,38,39]. Studies on mice models have confirmed that the lack of CD4^+^ T cells reduces the joint pathology of mice when infected with CHIKV [39]. [However, there are a very limited number of studies conducted using human patients to investigate the involvement of CD4 T cells in chronic CHIKV. The first human trial of the CHIKV-ChAdOx1 (candidate simian adenovirus vectored vaccine) showed induced CD4^+^, an IFNγ-biased T cell response against the CHIKV E1 and E2 proteins, and no significant CD8^+^ T cell responses [40].

Studies in human, non-human primates and mice models also show how CHIKV-infected macrophages may infiltrate and persist in tissues and that T-cell mediated adverse responses in joints could contribute to tissue damage and arthritis [36,41,42]. Specifically, CHIKV infection of CD14^+^ monocytic cells could infiltrate into the synovial cavity [36]. In mouse models infected by CHIKV, both CD4^+^ and CD8^+^ T cells have been shown to infiltrate into CHIKV-infected tissues [43,44]. However, the mechanism by which these cells contribute to the dual role of T cells in CHIKV infection remains unclear.

It is important to study the role of γδ T cells in CHIKV infection because γδ T cells are prevalent in the host subcutaneous area where the CHIKV is initially introduced via mosquito bite. These γδ T cells, lacking MHC complex restriction, are considered key players in cytotoxicity, cytokine secretion, and the induction of the maturation and function of dendritic cells [45,46]. One study conducted using mice model showed that mice infected with CHIKV had a higher number of γδ T cells in the infected foot and draining lymph node associated with higher production of proinflammatory cytokines and chemokines. Moreover, γδ T cell^−/−^ mice showed more signs of CHIKV infection, including greater foot swelling. This shows the protective role of γδ T cells in CHIKV infection at the early stage of the disease [47].

A recent preliminary study in humans suggests an association of peripheral regulatory T cells (T_regs_) and IL-10 with recovery from CHIKV infection. Higher secretion of IL-10 was observed in CHIKV-recovered individuals than in acute, chronic chikungunya arthritis patients and controls. The frequencies of T_regs_ and effector T cells (T_eff_) were higher, and the T_reg_/T_eff_ ratio was lower in rheumatoid arthritis (RA) patients than in chronic CHIKV arthritis patients. The results indicate that the reduction of T_regs_ is associated with ongoing CHIKV infection and the normalization of T_reg_ cells with resolution of the disease. Contrasting phenotypic data in RA and chronic chikungunya arthritis suggest an altogether different mechanism of T_reg_-mediated pathology in both arthritis conditions [48].

Although many studies have been conducted to determine the role of T cells in CHIKV infection with respect to CD4, CD8, γδ T cells, T_regs_, T_effs_ and macrophages, the exact role of T cell responses in human CHIKV infection and disease remains undefined. Moreover, the elucidation of the exact function of T cells is hampered by absence of comprehensive human studies and lack of knowledge of CHIKV-specific human T cell epitopes.

### 3.2. T Cell Response in DENV Infection

Many studies have been conducted to unravel the role of T cells in dengue infection over the past few decades. Higher activation of CD8^+^ T cells was recorded in acute dengue infection targeting NS3, NS4b and NS5 dengue viral antigens [49]. This initial CD8^+^ T cell activation helps to elicit cytotoxic damage by producing IFN-γ, perforin and granzymes, reducing the viral load. In contrast, CD4^+^ T cells target structural proteins of the virus i.e., capsid, envelope and secreted NS1 proteins, which are responsible for the production of memory T cells at the late stage of infection.

The role of CD8^+^ and CD4^+^ T cells in DENV is still being identified, along with whether it can provide protection during DENV infection or mediate a detrimental effect to the host by causing a cytokine-mediated immune pathology, resulting in a “cytokine storm” during a DENV infection. Several factors can determine this two-way role of T cells in dengue infection, such as the influence of prior dengue episodes, specific HLA-allele restriction, phenotypic differences of T cell expression during DENV and the regulation of gene expression with respect to specific T cell activation.

Studies have shown the protective role of CD8^+^ T cells in combating DENV infection through direct cytotoxicity and by the production of proinflammatory cytokines [15]. Skin, being the initial site for dengue viral invasion specifically through dermal antigen presenting cells, i.e., epidermal Langerhans cells, dermal dendritic cells and macrophages [50,51,52], could activate CD8^+^ T cells. The activation of CD8^+^ T cells plays an immunoprotective role during acute dengue infection, marking the front-line defense action of CD8^+^ T cells in dengue infection. Activation of peripheral CD8^+^ T cells during acute dengue infection upregulates the expression of several important chemokine markers, i.e., CCR5, CXCR3 and CXCR6 supporting the migration of T-cells into peripheral inflamed tissues. These CD8^+^ T cells also upregulate the expression of skin-homing cutaneous lymphocyte-associated antigen (CLA), which increases the influx of T cells to the skin of patients with acute dengue and provides immediate onsite protection [53,54]. After the recognition of viral peptides of 9-10 amino-acids in length through TCR, these activated antigen-specific CD8^+^ T cells then differentiate into effector cytotoxic T cells (T_eff_) in the secondary lymphoid organs to eliminate infected cells [55]. Cross-reactive DENV-specific CD8^+^ T cells can modulate the genetic expression of vascular endothelial growth factor receptor 2 (VEGFR2), contributing to vascular permeability in DENV infection [56].

It has been proposed that cross-reactive T cells raised against the original infecting serotype dominate during a secondary heterologous infection, a phenomenon that has been termed “original antigenic sin” [57,58]. In the context of dengue, this hypothesis postulates that during secondary infection, the expansion of pre-existing, lower avidity and the cross-reactive memory T cells dominate and induce a response qualitatively different from the response induced by the original antigen, inducing a “cytokine storm” which contributes to immunopathogenesis of severe forms of dengue disease, such as severe dengue [57]. Some studies have shown that infection with homologous DENV serotypes also reactivated memory T cells, leading to protection and increasing the DENV-specific antibody response [59,60]. During heterologous DENV infection, naïve and serotype cross-reactive memory T cells are activated, and a higher expansion of plasmablasts is observed. This expansion elicits stronger T cell responses, which help to activate memory B cells that produce large amounts of virus-specific IgG antibodies [61]. These memory B cells also show cross-reactivity for multiple serotypes [62,63]. However, these heterologous cross-reactive memory T cell activations are confined to certain HLA-restricted complexes. According to the original antigenic sin theory, the skewing of T cell responses induced by primary infection with one serotype would cause a less effective response upon secondary infection with a different serotype, predisposing individuals to severe disease. However, though skewing of responses toward primary infecting viruses was detected, this was not associated with an impairment of responses either qualitatively or quantitatively. Furthermore, HLA alleles associated with decreased susceptibility to severe disease were associated with responses of higher magnitude and greater polyfunctionality, suggesting that a vigorous response by multifunctional CD8 T cells is associated with protection from dengue virus disease [64]. Therefore, it is very important to investigate the role of memory T cells during homologous and heterologous DENV infection, as it is crucial in the development of effective DENV vaccines.

HLA alleles also influence the magnitude of both CD8 and CD4 T cell response in DENV infection. Some alleles are associated with weak CD4 and CD8 T cell responses. Conversely, some HLA alleles exert strong, multifunctional T cell responses and correlate with alleles associated with protection from severe disease both in human and animal models [64,65,66,67,68,69]. HLA-A*1101-restricted NS3_133-142_ represents one of the best-characterized T-cell epitopes for DENV and have been confirmed by many studies. A subset of HLA-B*35:01 DENV-specific T cells expressed programmed death 1 protein (PD-1), which may be a regulator to prevent excessive damage to the host by preserving the antiviral effects of the cells [70,71].

Studies have been conducted to explore the phenotypes of CD8^+^ T cells and their transcriptomic profiles during DENV infection. HLA-DR^+^CD38^+^ and HLA-DR^−^CD38^+^ CD8^+^ T cell subsets are highly abundant in dengue patients, upregulating genes with T cell activation, proliferation, migration and cytotoxicity [72]. Moreover, IFN-γ-producing CD8 T_em_ and T_emra_ subsets were also identified in PBMCs stimulated with DENV-derived peptides ex vivo, expressing upregulated genes for CD8 T cell activation, co-stimulation and other effector functions [73]. Follicular CXCR5^+^CD8^+^ T cells showed protective antiviral effects in DENV-2-infected patients [74]. Another study showed that high levels of IFN-γ^+^TNF^+^ CD8^+^ T cells were associated with mild dengue infection, and the response was higher to E and NS3 proteins compared to severe dengue infection [75].

CD4^+^ T cells have a more diverse function in developing efficient antibody response in B cells and helping CD8 T cells to elicit memory responses by differentiating into T helper type 1 (Th1) and follicular helper T (T_FH_) cells [76]. These CD4 T cells also have a cytotoxic effect by producing IFN-γ [77,78,79]. Recent studies have highlighted the protective role of CD4^+^ T cells in DENV infection [80]. For example, dengue-specific-HLA-DRB1 is associated with mild DENV infection [65]. In this context, type-I IFN plays an important role, activating DC. In turn, DC activation elicits immunity in specific subsets of T cells, i.e., CD45RA^+^CCR7^−^ effector memory re-expressing T cells (T_emra_), specifically by activating highly polarized CX3CR1^+^ cytotoxic CD4^+^ T cells. These CX3CR1^+^ cytotoxic CD4^+^ T cells play a protective role in DENV infections with a HLA DR allele [81]. Regulatory T cells (T_regs_) are another population of CD4 T cells which could expand in acute dengue to suppress highly cross-reactive DENV-specific T cells contributing to disease pathogenesis. Furthermore, studies have shown a high population of CD4^+^CD25^+^FoxP3 T_reg_, considered as a subset of T_reg_ in DENV patients in acute stage. This subset of CD4 T cells is untraceable at the convalescent stage [82,83].

Moreover, T_FH_ cells found in secondary lymphoid organs are important in helping B cells to develop and differentiate. These T_FH_ cells are also helpful in producing efficient pathogen-specific antibodies, which were found expanded in both human and mice during heterologous and homologous DENV infection, respectively, by possibly promoting the maturation of DENV-specific antibodies [84,85]. Peripheral T_FH_ cells (CXCR5^+^CD45RA^−^CD4^+^T cells) expand during the acute DENV infection, and these T_FH_ cells are activated during the critical phase of DENV infection, expressing high levels of PD-1 and CD38 [85]. These activated T_FH_ can express CD40L, which is considered a strong activator of B cells [86,87]. Another study showed that these T_FH_ cells are also activated when naïve CD4 T cells are co-cultured with DENV-infected dendritic cells [88,89].

### 3.3. Role of T Cells Response in ZIKV Infection

ZIKV infection is usually a mild or self-limiting infection and recently emerged as a major public health issue primarily in Southeast Asia and the Americas [90]. The main mode of transmission is through *Aedes* mosquitos. The virus can also transmit by sexual contact [91], making the ZIKV unique from other arboviral infections. The majority of ZIKV-infected patients remains asymptomatic and may develop mild Zika infection with rash, fever, arthralgia and conjunctivitis. However, in some cases, this infection further marks its uniqueness, causing serious complications in the central nervous system (CNS) that result in microcephaly during fetal development, congenital malformations and Guillain-Barre syndrome (GBS) [92,93,94]. In non-human primates, ZIKV can persist for roughly 10 days in plasma. After the first ZIKV clearance from the blood, the virus could also have prolonged detection in certain body fluids such as the cerebrospinal fluid (CSF) [95]. The type I IFN-γ signaling pathway is understood to play a protective mechanism which hinders flavivirus replication in the host at an early stage of infection, making the innate resistance essential for infection control. In ZIKV infection, the NS5 protein could inhibit type I IFN-γ signaling in human cells by targeting the IFN-regulated transcriptional activator STAT2 by proteasome-mediated degradation of STAT2. However, this phenomena has not been observed in murine models [96,97,98]. Therefore, it is important to use type I IFN-deficient mice (*ifnar*^−/−^) to study the innate immune responses for ZIKV infection in murine models [99,100].

Although the T cell immune profile of DENV infection has been widely studied, the number of studies available for ZIKV infection is limited. A recent study published by our team described an in-depth characterization of the immune profile of CD8^+^ T cell responses in ZIKV infection using both flow cytometry and transcriptomic methods [101]. This comprehensive study clearly shows that ZIKV-specific CD8^+^ T cell responses are predominately associated with IFN-γ responses, and this identification was made at the mRNA and protein level. This study also reported a set of specific genes highly associated with only CD8^+^-IFN-γ^+^ T cells which were significantly upregulated. These upregulated genes encoded proteins such as granzyme B, cytotoxic and regulatory T cell molecular (CRTAM), X-C motif chemokine ligand 1 and 2, and chemokine (C-C motif) ligand 3 and 4, which exerted different functions categorized into cytotoxicity, T cell activation and regulation, proinflammation, polyfunctional cytokines, and T cell homing. Another study from our team showed that ZIKV-specific CD8 T cell responses could be characterized by the release of IFN-γ, TNFα and granzyme B [17]. Cytokine profiles of acute and convalescent ZIKV patients were analyzed, and the resulting elevated cytokine profiles in acute ZIKV infection were associated with Th1, Th2, Th17 and Th9 responses. The levels of cytokines decreased during the recovery period [102].

CD8^+^ T cells are thought to play a protective role in ZIKV infection along with the high antibody response, allowing long-term resistance to ZIKV infection. A large number of studies have been conducted to determine the role of CD8 T cells in ZIKV in murine models. However, human-related studies are scarce [103,104,105,106,107]. It was shown that mice lacking CD8^+^ T cells challenged with ZIKV infection resulted in higher ZIKV titers in serum and tissues [103,104]. Further confirming this phenomena, a study showed that immunizing *ifnar*^−/−^ HLA-transgenic mice (i.e., HLA-B*07/02 and HLA-A*0101) with immunodominant peptides elicited a higher CD8 T cell response with lower ZIKV titers in the serum and brain, highlighting the role of CD8^+^ T cells in the neuropathology of ZIKV infection [108]. High titer of ZIKV in the fetal brain tissues and cerebrospinal fluid (CSF) was observed in other studies, highlighting the neurotropism of ZIKV [109]. Mice with Ifnar1 deficiency in the hematopoietic compartment are vulnerable to the spread of ZIKV from the site of infection to the brain, and Infar-deficient non-hematopoietic cells are responsible for the spread of ZIKV within the CNS. ZIKV-infected astrocytes break down the blood–brain barrier and allow the influx of CD8^+^ effector T cells, which plays a role in the neurological complications of ZIKV infection [110].

Both CD4^+^ and CD8^+^ cells are activated in many flavivirus infections, including ZIKV infection, which can exert inflammatory responses to the host. These inflammatory studies have been extensively studied in both human and non-human models [105,111,112]. Specifically, in ZIKV infection, these activated CD4^+^ T cells could further differentiate into effector memory and terminally differentiated T cells, enhancing the production and acquisition of cytokines. In addition, there is a significant reduction of IFN-γ-producing CD4 T cells in acute ZIKV patients [113]. In contrast to this human-based ZIKV study, another murine model study showed the activation of a higher percentage of IFN-γ-producing CD4^+^ T cells associated with reduction of ZIKV titers in CSF [114]. Moreover, the expansion of effector Vδ2 T cells in the acute phase of ZIKV infection was also observed, particularly expressing granzyme B [113]. These Vδ2 T cells play an important role in direct antiviral and stimulatory activities not only in ZIKV infection but also in many other acute viral infections [115,116].

Table 1 summarizes the dominant and activated T cell populations of CHIKV, DENV and ZIKV infection at different disease stages, and the respective production and upregulation of chemokine/cytokine.

## 4. The Impact of Sequence Homology and Cross-Reactivity between DENV, ZIKV and CHIKV Shaping Their T Cell Responses

Heterologous immunity in arboviral infections is an important area of interest, as many arboviral infections share similar geographical distribution and sequential or co-infections with different viruses can occur. The issue of potential ZIKV and DENV cross-reactivity and how pre-existing DENV T cell immunity modulates ZIKV T cell responses is of relevance as the two viruses often co-circulate. Moreover, ZIKV has been spreading in geographical regions where DENV is endemic, allowing ZIKV-infected individuals harboring prior DENV-reactive memory T cells to be infected [6,118,119]. The importance of studying this cross-reactivity between DENV and ZIKV infections would help to unravel many possible factors that may play a role in the immune response against the virus and in vaccine development. In this context, it is important to study how previous viral infection could affect the current heterotypic viral infection and the sequence homology between these various viruses, and to investigate whether there are specific conserved regions of these co-infected viruses. Many studies have confirmed serological cross-reactivity between DENV and ZIKV where antibodies developed against previous DENV have enhanced the protective immune response in current ZIKV infections [22,120,121,122,123,124,125,126,127]. In silico analysis has been used to predict cross-reactive ZIKV T cell epitopes based on sequence homology, and recent human data demonstrated that T cell immunity to ZIKV targets immunodominant epitopes that show cross-reactivity with other flavivirus in both humans [17] and HLA-transgenic mice [108,128]. Many studies have been conducted to determine how previous dengue infection changes the T cell response in current dengue infection with different serotypes [15,79].

A recent study mapped 93 human ZIKV T cell epitopes cross-reactive with DENV using in vitro expanded T cells taken from DENV-exposed, ZIKV-unexposed donors. These epitopes span through the ZIKV proteome, reporting high immunodominance specifically in the NS5 (25 epitopes) and NS3 (16 epitopes) regions, both accounting for 48% of total response. The reported epitopes are strongly associated with antigen size and the sequence identity of DENV [24].

The DENV/ZIKV cross-reactive CD8^+^ T cell response was also investigated using Ifnar-/- HLA -B*0702 and HLA-A*0101 transgenic mouse models. These mice were infected with DENV-2, and isolated PBMCs were treated with 37 ZIKV peptides and 14 HLA-B*0702-restricted ZIKV/DENV cross-reactive epitopes were found. The ZIKV-specific and ZIKV/DENV cross-reactive CD8^+^ T cell response was comparatively weak in prior DENV2-infected mice compared to primary ZIKV-infected mice. These depleted CD8^+^ T cells in prior DENV2-infected mice resulted in higher ZIKV infection, emphasizing that prior DENV immunity can change the specificity and magnitude of the CD8^+^ T cell response to subsequent ZIKV infection [108,129].

Memory T cell responses elicited by prior infection with DENV, or vaccination with an experimental tetravalent dengue live attenuated vaccine (TDLAV), recognize ZIKV-derived peptides identical or highly conserved in DENV and ZIKV, marking the first human characterization of both ZIKV specific and ZIKV/DENV cross-reactive T cell responses [17]. DENV exposure prior to ZIKV infection also influenced the timing and magnitude of the T cell response. ZIKV-reactive T cells in the acute phase of infection were detected earlier and in greater magnitude in DENV-immune patients. The quality of responses was also influenced by previous DENV exposure, and ZIKV-specific CD8 T cells from DENV pre-exposed donors with selectively upregulated granzyme B and PD1. The average sequence proteome homology between ZIKV and DENV was defined as 56%, and average homology of responding epitopes between DENV and ZIKV was defined as 77%. This further confirms the strong association between the sequence homology and cross-reactivity of DENV and ZIKV infection [17]. As described in the previous section, many studies have shown the role of T cell responses in heterotypic DENV infections resulting in “original antigenic sin,” and the phenomena is similar to the subsequent infection of DENV and ZIKV [130]. In line with these observations, studies have also shown stronger cross-reactive IFN-γ responses, specifically against the ZIKV-NS5 protein in Colombian donors [18] and the NS3 protein in West-African [131] and Singapore donors [20] who were previously infected with DENV. Brazilian patients who received live, attenuated tetravalent vaccine TV003 also showed high IFN-γ response upon ex vivo stimulation with ZIKV peptides [21].

Another study demonstrated that DENV/ZIKV co-infection decreased the ability of CD4^+^ T cells to produce IFN-γ^+^ and TNF^+^ compared to single DENV and ZIKV infections, showing the lack of cross-reactivity between DENV and ZIKV specifically CD4^+^ cells for envelope and NS1 proteins [17,121].

A recent study demonstrated that rhesus macaques (*Macaca mulata*) infected with ZIKV for 10 months followed by DENV infection showed early activation of CD4^+^ and CD8^+^ effector memory T cells, specifically recognizing CD4^+^ specific-DENV E protein eliciting higher levels of IFN-γ. Therefore, this study suggests that high DENV-E protein-reactive IFN-γ producing CD4^+^ T cells could exert a robust antibody response against future ZIKV and DENV infections [132].

After the ZIKV epidemic in Brazil and most of the Latin American and Caribbean countries in 2017, DENV cases decreased, providing evidence for studying whether prior ZIKV infection could modulate current DENV infection [133,134,135]. The influence of the ZIKV epidemic on the outbreak of DENV could play a complex role. A comprehensive study by Leah et al. (2020) suggests that ZIKV infection could increase the future risk of DENV infection [136]. Prospective pediatric cohorts in Nicaragua who experienced epidemics of DENV 1-3 from 2004 to 2015, ZIKV from 2016 to 2017 and DENV2 from 2018 to 2020 were used for this study. The results revealed that children with prior ZIKV infection had a 12.1% risk of developing dengue, which was significantly high compared to flavivirus-naïve children (3.5%) [136]. The potential increase of ZIKV pathogenesis in the presence of pre-existing dengue immunity has also been widely studied using human and non-human primates [137,138]. The involvement of DENV cross-reactivity in subsequent ZIKV infection and vice versa has been widely studied in the context of its clinical presentation, neutralizing antibodies and the duration between each infection [136,137]. Therefore, these studies further confirm the importance of investigating the role of CD8+ and CD4+ T cells in DENV/ZIKV cross-reactivity, which is imperative in future vaccine development against both DENV and ZIKV infections.

**Figure 4:**
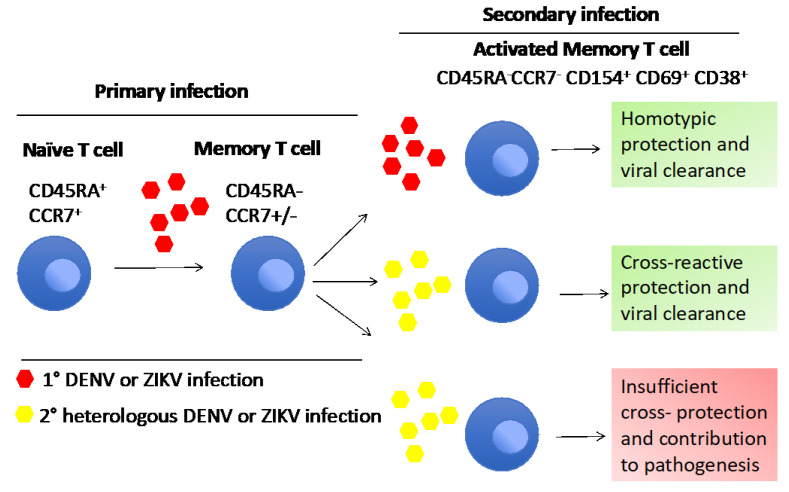
The dual role of T cells in flavivirus infection.

Homologous and heterologous viral cross-reactivity could be influenced by many factors, such as unique antigen presentation histories of each individual, genetic differences in HLA and TCR, virulence of the viral strain and many other unknown individual factors which could affect the final outcome [139]. So far, no studies have shown evidence for the cross-reactivity between DENV/CHIKV or ZIKV/CHIKV. A possible reason for the lower cross-reactivity between DENV/ZIKV and CHIKV is the lower level of genetic relatedness, as they belong to different viral families. Figure 4 explains the dual role of cross-reactive T cell response in flavivirus infections.

## 5. Current Understanding of T Cell Responses on Future Vaccine Development and Immune Therapy in Arboviral Infections

It is reasonable to assume that a successful arbovirus vaccine should induce durable adaptive B and T cells capable of inhibiting viral replication and dissemination. As reviewed above, a protective role for T cell responses has been implicated in DENV infection. A major drawback in the vaccine development of DENV infection is the unique mechanism of the dengue virus immune response, where secondary infection of the heterotypic serotype can cause higher risk of severe disease by enhancing viral entry and replication by non-neutralizing antibodies produced due to the prior primary DENV infection—a phenomena called “antibody dependent enhancement (ADE)” [140,141]. Therefore, for a given DENV vaccine, it is essential to consider the acquisition and coverage of all four serotypes to avoid this detrimental viral strategy. The first licensed dengue vaccine, Dengvaxia^®^, by Sanofi Pasteur, is a recombinant chimeric live attenuated DENV vaccine (CYD-TDV), harboring the 17D Yellow Fever non-structural backbone [9]. However, recipients of this vaccine who were DENV-naïve developed a high risk of hospitalization compared to unvaccinated controls [142,143]. Previous data clearly demonstrate that CD4 and CD8 T cell responses dominantly target non-structural proteins NS3, NS4B and NS5 [65,144]. Since these non-structural DENV proteins are absent in the recombinant dengue-Yellow Fever chimeric virus vaccine (CYD23) [145], our results explain the low level of vaccine efficacy observed. Another dengue live attenuated tetravalent vaccine (DLAV) was developed very recently and it showed a rapid increase of IFNg^+^TNF-α^+^-producing CD4^+^ T cells and then CD8^+^ T cells within 8–14 days after vaccination. These cells were detectable for at least 6 months. Vaccine-induced T_emra_ cells are detectable even after 1 year of vaccination, showing the T cell protection of this novel DENV vaccine [146]. Therefore, the assessment of T cell responses in concert with antibody responses is of key interest in the context of vaccine development. Our studies identified and characterized the epitopes recognized by the tetravalent DENV vaccine. These studies showed that the responses resemble the T cell responses observed in the context of natural infection and show limited cross-reactivity with other flaviviral infections [23,147,148].

Several promising ZIKV and CHIKV vaccine candidates are being tested in human clinical trials. These vaccines rely on diverse technologies, spanning from the use of live attenuated viruses or replication-defective viruses to nucleic acid-based vaccine or vectors expressing only certain viral proteins [149]. The observation that a primary virus infection rhesus macaque inhibited a secondary challenge with a homologous or heterologous virus suggests that anti-ZIKV adaptive immunity can be protective, and early-phase clinical trials have demonstrated safety and immunogenicity [150,151]. However, vaccines associated with weak or absent T cell responses could maximize the potential enhancement of DENV infection. Conversely, T cell responses that cross-react with self-components could play a role in the induction of GBS due to ZIKV infection [53,107,152,153]. The role of the cross-reactive T cells between DENV and ZIKV on each vaccine deliverable is also a major concern [154,155]. Thus, it is important to understand the ZIKV and CHIKV vaccine-induced T cell epitopes and their associated phenotypes in comparison to natural ZIKV and CHIKV infections. Moreover, validation of these epitopes according to HLA class I and Class II restriction is also important to evaluate the potential vaccine candidates and to allow the comparison of vaccine performance with field studies to examine natural immunity.

## Figures and Tables

**Table 1:** The role of T cell populations of CHIKV, DENV and ZIKV during different disease stages, and their respective function, gene expression and regulation profile.

Virus	T Cell Type/Phenotype	Disease Condition	Markers	Gene Regulation	Function	References
CHIKV	CD8^+^ T cells	Acute	CD69, CD107a, Granzyme B, Perforin, IFNγ	Upregulation	T cell activation, destroy virus infected cells	[32,34,35,36]
		Acute	IL-17, IL-10 R	Downregulation	develop joint symptoms in acute phase and maintain these symptoms in the chronic phase	[35]
		Chronic	CD95L	Upregulation	involved in CHIKV chronicity mechanism	[35]
	CD4^+^ T cells	Acute	CD95L	Upregulation	apoptosis of CD4 T cells	[32]
		Chronic	IFNγ,	Upregulation	inflammation and joint swelling	[39]
	γδ T cells	Acute	CCL2, CXCL9, IFNγ	Downregulation	reduced foot swelling in mice	[45,46,109]
	CD4-T_reg_	recovery	IL-10	Up regulation	resolution of the disease	[48]
DENV	CD8^+^ T cells	Acute	CCR5, CXCR3, CXCR6	Upregulation	supporting T cells to migrate in to peripheral inflamed tissues	[53,54]
			CLA	Upregulation	influx of T cells to skin providing immediate onsite protection	[117]
			CD69, HLA-DP, DQ, DR, CD38, cytotoxic granule TIA-1, VLA-4, ICAM-1, LFA-1CD44, CD11a	Upregulation	T cell activation, elimination the virus, induce inflammation	[117]
	HLA-DR^+^CD38^+^ CD8^+^T cells	Acute	PD-1, Lag3, KLRG1, CTLA-4, CD160	Upregulation	T cell activation, Proliferation, cytotoxicity and migration	[72]
	HLA-DR^−^CD38^+^ CD8^+^ T cells	Acute	AKT3, ACTN1	Downregulation	TCR signaling, amplification and synapse	[72]
	CD8^+^T_em_ (CD45RA^−^CCR7^−^) and T_emra_ (CD45RA^+^CCR7^−^)	Acute	IFNγ, CCL3/CCL4, CD69, CRTAM, IFNγ, TNFα, CTLA4, ICOS, LIGHT, IRF4, IRF8, SLAMF7, KIR2DL3	Upregulation	T cell activation, proliferation and polyfunctional properties, narrow transcriptional responses	[73]
	CXCR5^+^CD8^+^ T cells (T_FH_)	Chronic	PD-1	Upregulation	T cell proliferation and exert effector functions	[74]
	CD4^+^ T cells		IFNγ	Upregulation	cytotoxic effects	[77,79]
	CD4^+^Temra CD45RA^+^CCR7^−^		CX3CR1, serine protease granzyme, IFNγ	Upregulation	cytotoxic, protective role in DENV with HLA DR allele	[81]
	CD4^+^CD25^+^FoxP3^+^ T_reg_	acute	CTLA-4	Upregulation	producing immunosuppressive cytokines	[82,83]
	T_FH_	critical	CD40L	Upregulation	strong activator of B cells	[86,87]
	T_FH_CXCR5^+^CD45RA^−^CD4^+^Tcells	Critical	PD-1, CD38	Upregulation	predictor for neutralizing antibody titer and disease severity	[85]
ZIKV	CD8^+^IFN-γ^+^ T cells	acute	granzyme B, CRTAM, X-C motif chemokine ligand 1 and 2, CC3, CC4	Upregulation	cytotoxicity, T cell activation and regulation, proinflammation, T cell homing	[101]
	CD8^+^IFN-γ^+^ T cells	Acute	IFNγ, TNF-α, Granzyme B	Upregulation	Th1, Th2, Th17 and Th9 responses	[17,102]
	Vδ2 T cells	Acute	Granzyme B	Upregulation	antiviral and stimulatory activities	[115,116]

## Data Availability

Not applicable.
